# Comparative and Temporal Characterization of LPS and Blue-Light-Induced TLR4 Signal Transduction and Gene Expression in Optogenetically Manipulated Endothelial Cells

**DOI:** 10.3390/cells12050697

**Published:** 2023-02-22

**Authors:** Anna Stierschneider, Benjamin Neuditschko, Katrin Colleselli, Harald Hundsberger, Franz Herzog, Christoph Wiesner

**Affiliations:** 1 Department of Medical and Pharmaceutical Biotechnology, IMC University of Applied Sciences, 3500 Krems, Austria; 2 Institute Krems Bioanalytics, IMC University of Applied Sciences, 3500 Krems, Austria

**Keywords:** endothelial cells, Toll-like receptor 4, lipopolysaccharide, optogenetic control, pro-inflammatory proteins, quantitative mass-spectrometry, chemotaxis, transmigration

## Abstract

In endothelial cells (ECs), stimulation of Toll-like receptor 4 (TLR4) by the endotoxin lipopolysaccharide (LPS) induces the release of diverse pro-inflammatory mediators, beneficial in controlling bacterial infections. However, their systemic secretion is a main driver of sepsis and chronic inflammatory diseases. Since distinct and rapid induction of TLR4 signaling is difficult to achieve with LPS due to the specific and non-specific affinity to other surface molecules and receptors, we engineered new light-oxygen-voltage-sensing (LOV)-domain-based optogenetic endothelial cell lines (opto-TLR4-LOV LECs and opto-TLR4-LOV HUVECs) that allow fast, precise temporal, and reversible activation of TLR4 signaling pathways. Using quantitative mass-spectrometry, RT-qPCR, and Western blot analysis, we show that pro-inflammatory proteins were not only expressed differently, but also had a different time course when the cells were stimulated with light or LPS. Additional functional assays demonstrated that light induction promoted chemotaxis of THP-1 cells, disruption of the EC monolayer and transmigration. In contrast, ECs incorporating a truncated version of the TLR4 extracellular domain (opto-TLR4 ΔECD2-LOV LECs) revealed high basal activity with fast depletion of the cell signaling system upon illumination. We conclude that the established optogenetic cell lines are well suited to induce rapid and precise photoactivation of TLR4, allowing receptor-specific studies.

## 1. Introduction

Endothelial cells (ECs) line the inner wall of blood vessels and are located at the interface between circulating blood and the surrounding tissue. Thus, endothelial cells are the first cells exposed to invading pathogens circulating in the blood stream. The endotoxin lipopolysaccharide (LPS) is derived from the outer cell wall of gram-negative bacteria and triggers endothelial activation through a receptor complex consisting of the Toll-like receptor 4 (TLR4), CD14, and MD2 [1]. Subsequent recruitment of the adaptor proteins Toll/interleukin 1-receptor (TIR)–domain-containing (TRIAP) and myeloid differentiation factor (MyD88) initializes the MyD88-dependent pathway leading to early activation of the nuclear factor κB (NF-κB) and the mitogen-activated protein kinases (MAPK) [2,3]. Sequential binding of the TIR-domain-containing adaptor-inducing interferon-β (TRIF) and the TRIF-related adaptor molecule (TRAM) to the TIR domain of TLR4, and the subsequent dynamin-controlled endosomal translocation of TLR4, characterize the MyD88-independent pathway, which culminates in a late-phase activation of NF-κB [4]. The final production of various pro-inflammatory mediators is beneficial in controlling bacterial infections; however, their systemic secretion is a main driver of sepsis and chronic inflammatory diseases [5,6]. The molecular and regulatory mechanisms of LPS/TLR4-induced signaling events have been extensively studied in recent years, accelerating the identification of negative regulators of LPS signaling cascades [7,8,9]. Common strategies for studying physiological processes in inflammation often rely on genetic manipulation of the proteins under study, or the treatment of cells with agonists or antagonists. However, these strategies often lead to irreversible phenotypes in the target cells, or to unintended cytotoxicity and signaling crosstalk due to off-target or pleiotropic effects. Using light to manipulate signaling processes in living cells is one of the most elegant techniques developed in recent years. It involves integrating light-sensitive protein domains of photoreceptors into effector proteins in order to direct them with light stimuli in a spatiotemporal and minimally invasive manner [10]. A multitude of optogenetic switches have already been designed to control the activation, inactivation, localization, stabilization, or destabilization of signaling pathways [11]. In this study, we fused the light-oxygen-voltage (LOV)-sensing domain, isolated from the yellow-green algae *Vaucheria frigida aureochrome 1*, C-terminally to the full-length TLR4, as well as to different versions in which the extracellular domain of TLR4 was deleted (ΔECD), in order to enable blue-light-induced homodimerization and subsequent activation of TLR4-related pro-inflammatory signaling pathways [12,13]. Since this photoreaction is reversible, endothelial cells with stable integrated TLR4-LOV constructs allow a very specific investigation of the underlying molecular and regulatory mechanisms of the receptor with spatial and temporal precision.

## 2. Materials and Methods

### 2.1. Cells and Cell Culture

The 293Ta (GeneCopoeia^TM^, Rockville, MD, USA; LT008) (RRID: CVCL_BT05) were maintained in a DMEM growth medium (Thermo Fisher Scientific, Vienna, Austria; 31053044) supplemented with 100 U/mL penicillin/streptomycin (Thermo Fisher Scientific, Vienna, Austria; 15140-122), 2 mM L-glutamine (Thermo Fisher Scientific, Vienna, Austria; 25030-24) and 10% heat-inactivated fetal calf serum (Thermo Fisher Scientific, Vienna, Austria; A5256801). Human lymphatic endothelial cells (LECs) immortalized by ectopic expression of telomerase reverse transcriptase [14], human umbilical vein endothelial cells (HUVECs) (provided by the Medical University of Graz), and primary peripheral blood mononuclear cells (PBMCs) (ATCC^®^, Manassas, VD, USA; PCS-800-011™) (RRID:CVCL_WR41) were cultivated in a huMEC medium (InSCREENex, Braunschweig, Germany; INS-ME-1012) supplemented with 100 µg/mL Normocin (InvivoGen, Toulouse, France; ant-nr-1). LECs with stable integrated NF-κB-Gluc reporters and stable integrated TLR4-LOV or TLR4 ΔECD2-LOV constructs were cultivated with 100 µg/mL hygromycin B (Thermo Fisher Scientific, Vienna, Austria; 10687010) and 1 µg/mL puromycin dihydrochloride (Thermo Fisher Scientific, Vienna, Austria; A1113803), respectively. THP-1 (ATCC^®^, Manassas, VD, USA; TIB-202) (RRID: CVCL_0006) was cultivated in RPMI-1640 (Thermo Fisher Scientific, Vienna, Austria; 32404014), supplemented with 100 U/mL penicillin/streptomycin, 2 mM L-glutamine, and 10% fetal calf serum (Thermo Fisher Scientific, Vienna, Austria; 10270106). Monocytes (THP-1) were differentiated into monocyte-derived macrophages (THP-1 M0) by adding 4 ng/mL phorbol-12-myristat-13-acetate (PMA) (THP, Vienna, Austria; HY-18739) to a complete medium for 24 h and incubating for a further 24 h in a complete medium without PMA. Cells were passaged when a confluency of 90% was reached. For detachment of adherent cells, 0.25% Trypsin-EDTA (Thermo Fisher Scientific, Vienna, Austria; 25200-056) was used. Cell culture flasks (Sarstedt, Nümbrecht, Germany; 83.3911.002) for the maintenance of ECs were pre-coated with 0.5% gelatin (InSCREENex, Braunschweig, Germany; INS-SU-1015).

### 2.2. Engineering Light-Oxygen-Voltage-Based Optogenetic TLR4 Constructs and NF-κB Reporter

The cloning strategy of the TLR4-LOV constructs and NF-κB-TRE-Gluc reporter used was performed as previously described [13]. TLR4 ΔECD2/15/21/36-LOV was engineered using the following primers: Forward: 5’-AATTTCGGATGGC-3’ (ΔECD2); 5’-TTCAATGGCATCTT-3’ (ΔECD15); 5’-TGGATCAAGGACCA-3’ (ΔECD21); 5’-ACACCTCAGATAAGC-3’ (ΔECD36); and reverse: 5’-[phos]CTTCTCAACC-3’ (ΔECD2/15/21/36).

### 2.3. Transfection

The 293Ta was transiently co-transfected with 7.5 µg of NF-κB-TRE-Gluc, Hygro reporter plasmid (THP, Vienna, Austria; CS-NF-κB-02) or AP-1 *cis*-Reporting System (Agilent Technologies, Santa Clara, CA, USA; 219073), and 7.5 µg of the engineered TLR4, TLR4-LOV, or TLR4 ΔECD2/15/21/36-LOV plasmid, respectively, in 10 cm Petri dishes using the calcium phosphate precipitation technique [15]. Then, 24 h later, cells were re-seeded and treated as described in Section 2.4. For stable transfection of engineered TLR4-LOV, TLR4 ΔECD2/15/21/36-LOV, and NF κB-TRE-Gluc, Hygro (THP, Vienna, Austria; CS-NF-κB-02), a lentiviral transfection system was performed as previously described [13].

### 2.4. Substance Treatment and Blue Light Stimulation

The 293Ta and ECs were seeded into 96-well plates (Sarstedt, Nümbrecht, Germany; 83.3924300) (NF-κB/AP-1 reporter assay), black 96-well plates with an optical bottom (Thermo Fisher Scientific, Vienna Austria; 165305) (p65 localization), 6-well plates (Sarstedt, Nümbrecht, Germany; 83.3920300) (real-time quantitative PCR, Western blotting) or 8-well chambers (ibid, Gräfelfing, Germany; 80841) pre-coated with 0.5% gelatin (InSCREENex, Braunschweig, Germany; INS-SU-1015) (TLR4 localization), and they were grown in a complete medium until they were confluent. Cells were starved in a complete medium supplemented with 2% fetal calf serum for 2 h. Conventional TLR4 activation was induced with 100 ng/mL LPS from *Escherichia coli 026:B6* (Thermo Fischer Scientific, Vienna, Austria; 00-4976-03). For light-induced TLR4-LOV activation, cells were exposed to blue light (470 nm, 8 V) using the Amuza LED assay system 10335 (San Diego, CA, USA). To block TLR4 signaling, cells were treated with 1, 10, or 100 µM TAK-242 (resatorvid; Merck, Darmstadt, Germany; 614316-5MG), whereas NF-κB inhibition was provoked by the addition of 0.3, 3, or 30 µM parthenolide (Abcam, Cambridge, United Kingdom; ab120849).

### 2.5. Gaussia Luciferase and Firefly Luciferase Reporter Gene Assay

NF-κB activation was quantified by measuring gaussia luciferase (Gluc) reporter using the Secrete-Pair^TM^ Gaussia Luciferase Dual Luminescence Assay Kit (THP, Vienna, Austria; LF062) according to the manufacturer’s instructions. AP-1 activation was determined by measuring firefly luciferase (Fluc) reporter using the Luc-Pair^TM^ Firefly Luciferase HT Assay Kit (THP, Vienna, Austria; LF018) according to the manufacturer’s instructions. Relative luminescence units (RLU) were measured in a plate reader (SpectraMaxi3x, Molecular Devices, LLC, San Jose, CA, USA; Luminescence Glow, Lum 384 cartridge), and normalized to the cell count generated with an imaging cytometer (Mini Max 300, Molecular Devices, LLC, San Jose, CA, USA).

### 2.6. Immunostaining for Fluorescence Microscopy

For TLR4 and p65 localization, EC monolayers were chemically fixed with 4% paraformaldehyde at room temperature for 30 min at the specified stimulation time points and permeabilized with 0.1% Triton-X-100 (Merck, Darmstadt, Germany; 11332481001) at room temperature for 15 min. ECs were gently washed with PBS and unspecific binding was blocked by incubation with 1% bovine serum albumin (Thermo Fisher Scientific, Vienna, Austria; 15260037) in PBS overnight at 4 °C. Subsequently, ECs were stained with primary antibodies anti-TLR4, h-Toll, CD284 (US Biological, Salem, MA, USA; 042879; 1 to 50 in PBS with 1% bovine serum albumin), or anti-NF-κB p65 antibody (Abcam, CA, USA; ab16502; 5 µg/mL in PBS with 1% bovine serum albumin) at room temperature for 2 h. After washing with PBS-T (0.05%), ECs were incubated with secondary antibody Alexa Fluor 488 goat anti-rabbit IgG (H+L) (Thermo Fisher Scientific, Vienna, Austria; A11008; 4 µg/mL in PBS-T (0.05%)) at room temperature for 1 h. Cell nuclei were stained with Hoechst 33342 (Thermo Fisher Scientific, Vienna, Austria; H1399; 2 µg/mL in PBS-T (0.05%)) at room temperature for 10 min before being washed with PBS-T (0.05%). For TLR4 localization, ECs were mounted with Roti^®^-Mount Fluor Care (Carl Roth, Karlsruhe, Germany; HP19.1) on high-precision microscope cover glasses (1.5H, Marienfeld, Lauda-Königshofen, Germany; 0107242). Fluorescent images were acquired with a confocal laser scanning microscope (TCS SP8, Leica, Mannheim, Germany) using a 63X glycerol objective (numerical aperture 1.3). Images were analyzed with the Leica Application Suite Version X 3.5.7.23225 software. For p65 localization, fluorescent images of ECs were directly taken from the 96-well plate with an inverted microscope (DMI6000 B, Leica, Mannheim, Germany) using a 63X objective and analyzed with the Leica Application Suite Version X 3.8.0 software. To quantify nuclear localization of NF-κB, the mean ratio of the fluorescence intensity of stained p65 in the nucleus to the cytoplasm was computed using ImageJ [16].

### 2.7. Real-Time Quantitative PCR (qPCR)

Total RNA was extracted and purified using the RNeasy^®^ Mini Kit (Qiagen, Vienna, Austria; 74104) and 1 µg RNA was reverse transcribed using the Hight Capacity cDNA Reverse Transcription Kit (Thermo Fisher Scientific, Vienna, Austria; 4368814) according to the manufacturers’ instructions. For target gene quantification following pre-designed TaqMan^®^ Gene Expression Assays, a pair of unlabeled PCR primers, a TaqMan^®^ probe with a FAM dye label on the 5’-end, a minor groove binder, and a nonfluorescent quencher on the 3’-end, were used: TLR4: Hs00152939_m1, IL-6: Hs00985639_m1, CXCL-8: Hs00174103_m1, CXCL-10: Hs00171042_m1, and IPO8: Hs00183533_m1 (control gene). The qPCR reaction mix contained a final volume of 10 µL of TaqMan^®^ Gene Expression Master Mix (Thermo Fisher Scientific, Vienna, Austria; 4369016), 1 µL of TaqMan^®^ Gene Expression assay, 5 µL of nuclease free water (Ambion, Austin, TX, USA; AM9937), and 4 µL of cDNA template diluted 1:10. All qPCRs were run on the Quant Studio 7 Flex (Applied Biosystems, Foster City, CA, USA; QSTUDIO7FLEX) using the following cycling conditions: 10 min at 95 °C for initial denaturation followed by 45 cycles of 95 °C for 20 s and 60 °C for 1 min. Data were analyzed using the Quant Studio Real-Time PCR Software v1.3 (Applied Biosystems, Foster City, CA, USA). Relative gene expression levels were calculated according to the comparative C_T_ method (2^-ΔΔCT^) [17]. The mRNA target gene expression levels were computed relative to the endogenous control gene (IPO8).

### 2.8. Western Blotting

Cells were washed once with PBS and collected in 100 µL ice-cold lysis buffer (500 mM NaCl (Merck, Darmstadt, Germany; S5150), 50 mM Tris-HCl pH 7.4, (Thermo Fisher Scientific, Vienna, Austria; 15568-025), 0.1% SDS (Carl Roth, Karlsruhe, Germany; 1057.1), 1% NP-40 (VWR, Radnor, PA; USA; M158), 1U DNase I (Thermo Fisher Scientific, Vienna, Austria; 89836), 1 U protease, and a phosphatase inhibitor cocktail (Thermo Fisher Scientific, Vienna Austria; 1860932, 78428)). Cell lysates were shaken for 20 min on ice, followed by centrifugation at 12,000 rpm for 20 min at 4 °C. The total protein concentration of the supernatant was determined using a BCA protein assay (Thermo Fisher Scientific, Vienna, Austria; 23227) according to the manufacturer’s manual. A 4x Laemmli sample buffer (Bio-Rad, CA, USA; 1610747) containing 10% ß-mercaptoethanol (Merck, Darmstadt, Germany; M7522) was added to 1 µg protein and incubated at 70 °C for 10 min. For the investigation of transmembrane proteins (TLR4), lysates must not be heated. Protein extracts were subsequently loaded onto 7.5% Mini-PROTEAN TGX Precast Protein Gels (Bio-Rad, CA, USA; 456-1023) and run at 100 V before protein bands were electro-blotted onto nitrocellulose membranes (Bio-Rad, CA, USA; 1704155) using the Trans-Blot^®^ Turbo^TM^ Blotting System (Standard, 35 min). Unspecific binding was blocked with 5% non-fat dry milk (New England Biolabs, Frankfurt, Germany; 9999) in PBS-T (0.1%) buffer on a shaker overnight at 4 °C. The primary and secondary antibodies used are specified in Table 1. Membranes were incubated with primary antibodies following secondary antibodies at the stated dilution and diluent at room temperature for 2 h each. Immunoblots were developed by applying the Clarity Western ECL Substrate (Bio-Rad, CA, USA; 1705060) according to the manufacturer’s instructions. Proteins were visualized with the chemiluminescence detector of the ChemiDoc MP platform (Bio-Rad, CA, USA; 17001402).

### 2.9. Quantitative Mass-Spectrometry

Opto-TLR4-LOV LECs were seeded in T25 cell culture flasks (Sarstedt, Nümbrecht, Germany; 83.3910.002) and grown in a complete medium until confluent. EC monolayers were subsequently washed twice with PBS and stimulated with LPS or blue light, or left untreated as described in Section 2.4, in 3 mL huMEC medium (InSCREENex, Braunschweig, Germany; INS-ME-1012) without fetal calf serum. After incubation at 37 °C for 2 h, 6 h, or 16 h, the medium was collected and the proteins contained were precipitated using cold ethanol (ROTIPURAN^®^ ≥99.8%, p.a., Carl Roth, Karlsruhe, Germany; 9065.1) and stored at −20 °C. After centrifugation at 3500 ×*g*, 4 °C for 30 min, the supernatant was discarded and the proteins were dried and dissolved in an 80 µL lysis buffer (8 M Urea, 50 mM NH₄HCO₃). After subsequent further centrifugation at 2000 ×*g* for 10 min, the supernatant was transferred and stored at −20 °C until further use. The protein concentration was determined using a BCA protein assay (Sigma-Aldrich, Vienna, Austria; 71285-3) according to the manufacturer’s instructions. A total of 20 µg of protein per sample was reduced and alkylated using TCEP and IAA and digested successively using Lys-C (FUJIFILM Wako Chemicals U.S.A. Corporation, Richmond, VA, USA; 125-05061) for one hour and Trypsin (Promega, Walldorf, Germany; V5113) for 16 h. Peptides were cleaned using Sep-Pak tC18 1 cc Vac Cartridges (Waters, Vienna, Austria; WAT054960), then dried and stored at −20 °C.

For HPLC-MS/MS analysis, samples were analyzed using an Ultimate 3000 RSLCnano system coupled to a Orbitrap Eclipse Tribrid mass spectrometer (both Thermo Fisher Scientific, Vienna, Austria). The dried samples were dissolved in 40 µL mobile phase A (98% H2O, 2% ACN, 0.1% FA) and measured in duplicates. A total of 2 µL was injected onto a PepMap 100 (C18 0.3 × 5mm) TRAP column and analyzed using a PepMap RSLC EASY-Spray column (C18, 2 µm, 100 Å, 75 µm × 50 cm, Thermo Fisher Scientific, Vienna; ES903). Separation occurred at 300 nL min-1 with a flow gradient from 2% to 35% mobile Phase B (2% H_2_O, 98% ACN, 0.1% FA) within 60 min, resulting in a total method time of 80 min. The mass spectrometer was operated with the FAIMS Pro System in the positive ionization mode at CV-45. The scan range was 375–1500 m z-1 using a resolution of 120,000 at 200 m z-1 on MS1 level. Isolated peptides were fragmented using HCD at a collision energy of 30% NCE and fragments were analyzed in LIT using rapid scan mode. For protein identification and quantification, FragPipe (v18.0) was used selecting the LFQ-MBR workflow, and employing MSFragger (v3.5) [18] and IonQuant (v1.8.0) [19]. For statistical evaluation, Perseus (v2.0.6.0) [20] was used. Measurement duplicates were averaged, and protein groups were filtered according to their treatment, demanding at least three out of four values to be valid in at least one group. The remaining missing values were replaced with a down shift of 1.8 and a width of 0.3 to allow statistical testing for all remaining protein groups. ClueGO application [21] in Cytoscape [22] was used to group proteins according to GO terms. The mass spectrometry proteomics data have been deposited to the ProteomeXchange Consortium via the PRIDE [23] partner repository with the dataset identifier PXD038764.

### 2.10. Chemotaxis Assay

The chemotactic response of THP-1 cells to the medium of LPS or blue-light-stimulated opto-TLR4-LOV LECs was performed using Transwell^®^ 96-well permeable supports (Merck, Darmstadt, Germany; CLS3388) and Transwell^®^ 24-well permeable supports (Szabo-Scandic, Vienna, Austria; COS3421). Thereafter, opto-TLR4-LOV LECs were seeded in T25 cell culture flasks (Sarstedt, Nümbrecht, Germany; 83.3910.002) and grown in a complete medium until confluent. EC monolayers were subsequently washed twice with PBS and stimulated with LPS or blue light, or left untreated as described in Section 2.4, in 3 mL huMEC medium (InSCREENex, Braunschweig, Germany; INS-ME-1012) without fetal calf serum. After incubation at 37 °C for 6 h, the medium was collected and applied to the lower chamber of the transwell plates (100 µL to the 96-transwell plates and 600 µL to the 24-transwell plates). THP-1 cells were stained with Hoechst 33342 (Thermo Fisher Scientific, Vienna, Austria; H1399, 2 µg/mL) at 37 °C for 10 min and resuspended at a density of 1,000,000 c/mL in a huMEC medium without fetal calf serum, then applied to the upper chamber of the Transwell plates (50 µL to the 96 permeable supports and 100 µL to the 24 permeable supports). Post-incubation at 37 °C for 2 h, the remaining THP-1 cells of the upper chamber that did not migrate through the filters of the permeable supports were aspirated. THP-1 cells attached on the upper side of the filter were gently removed with a cotton swab. THP-1 cells that migrated through the filters of the 96-well permeable supports were quantified by measuring relative fluorescence units with a plate reader (SpectraMaxi3x, Molecular Devices, LLC, San Jose, CA, USA; Fluorescence Intensity cartridge; excitation 350 nm, emission 461 nm) in a well scan manner, whereas THP-1 cells that migrated through the filter of the 24-well permeable supports were visualized with an inverted microscope (DMI6000 B, Leica, Mannheim, Germany) using a 40X objective and analyzed with the Leica Application Suite Version X 3.8.0 software.

### 2.11. Electrical Cell-Substrate Impedance Sensing Technology

An EC monolayer breakdown (transmigration) assay was performed using the electrical cell-substrate impedance sensing (ECIS) model 9600Z (Applied BioPhysics, Troy, NY, USA). Opto-TLR4-LOV LECs, opto-TLR4 ΔECD2-LOV LECs and opto-TLR4-LOV HUVECs were seeded at a density of 40,000 cells/100 µL of complete medium onto a 96-well plate containing 20 gold film electrodes per well (96W20idf PET; ibidi, Gräfelfing, Germany; 72098) pre-coated with 1 mg/mL neutralized rat tail collagen type I (Thermo Fisher Scientific, Vienna, Austria; A1048301) at room temperature for 10 min, and 2 µg/mL bovine plasma fibronectin (Thermo Fisher Scientific, Vienna, Austria; 33010018) at 37 °C for 45 min. A small-amplitude AC signal (4000 Hz) (I) was imposed across the electrodes, onto which cells were attached, resulting in a potential (V) across the electrodes that was measured using the ECIS instrument [24]. The impedance (Z) was determined by Ohm’s law Z=V/I. ECs were grown to confluent monolayers for 27 h before being treated with blue light, LPS (100 ng/mL), 50,000 c/well THP-1, 50,000 c/well THP-1 M0, 50,000 c/well PBMC, combinations of blue light or LPS, and the mentioned cell types, or left untreated. EC breakdown was assessed by continuous resistance measurement for 3 h.

### 2.12. Statistical Analysis

All experimental figures show data from at least two technical replicates, while *n* represents the number of biological replicates. The mean ± standard deviation of all data was computed and graphically displayed using GraphPad Prism version 9.3.0 for Windows (GraphPad Software, San Diego, California USA, www.graphpad.com). Post-ANOVA multiple comparisons relative to the control were performed using Dunnett’s or Šidák’s tests and they are specified in the figure legend. Probability values of *p < 0.05* were considered statistically significant.

## 3. Results

### 3.1. LPS and Blue Light Induces NF-κB and AP-1 Activation of Designed TLR4-LOV Constructs

**Figure 1:**
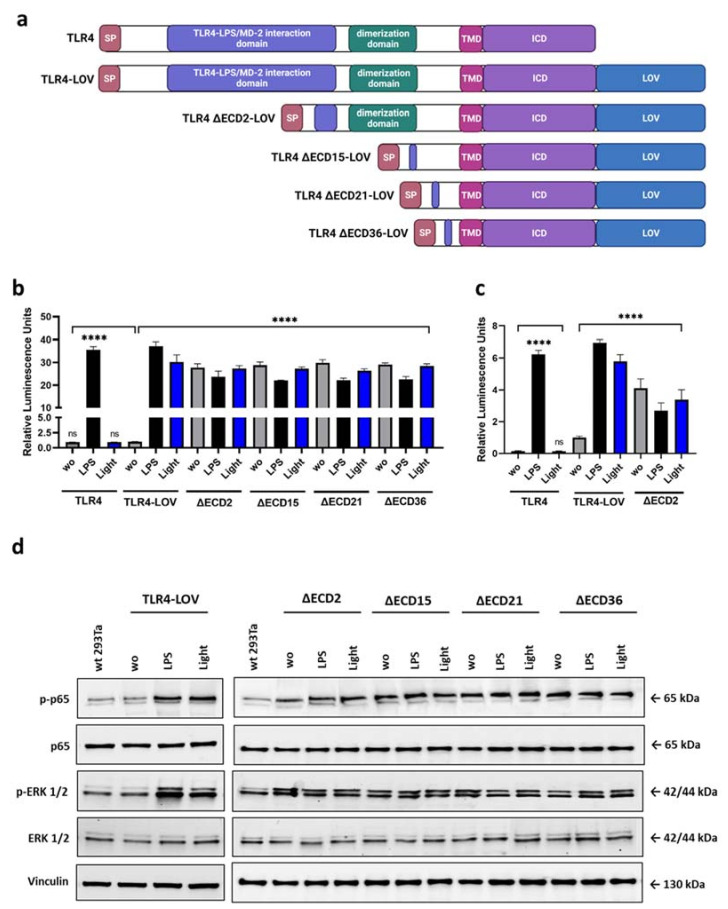
Design and function of TLR4-LOV constructs. (**a**) Schematic drawing of the full-length TLR4, TLR4-LOV and truncated TLR4 ∆ECD2/15/21/36-LOV constructs. Toll-like receptor 4 consists of an extracellular domain (signal peptide (SP), TLR4-LPS/MD2 interaction domain and dimerization domain), a transmembrane domain (TMD), and an intracellular domain (ICD). TLR4-LOV and deletion constructs comprise a photosensitive LOV domain fused to the C-terminus. TLR4 ∆ECD2-LOV contains a deleted LPS/MD2 interaction domain. TLR4 ∆ECD15/21/36-LOV are constructs with distinct LPS/MD2 interaction domain and dimerization domain deletions. (**b**,**c**) The 293Ta cells were transiently co-transfected with TLR4, TLR4-LOV, or TLR4 ∆ECD2/15/21/36-LOV with (**b**) NF-κB-Gluc or (**c**) AP-1 cis reporter for 24 h and luciferase activity measured 6 h post-LPS (100 ng/mL) or blue light (470 nm) stimulation, or no treatment. Activation is expressed as induction of (**b**) gaussia luciferase reporter gene (NF-κB) or (**c**) firefly luciferase reporter (AP-1) and normalized to the cell count. Mean values ± standard deviation were graphically displayed in bar charts (*n* = 6). Post-ANOVA, multiple comparisons relative to the control were performed using Šidák’s test (ns = not significant, **** *p* < 0.0001). (**d**) Western blot analysis of phospho-p65, p65, phospho-ERK1/2, and ERK1/2 expression 6 h post-LPS (100 ng/mL) or blue light (470 nm) stimulation, or no treatment of transient transfected 293Ta cells. Vinculin was used as loading control.

To investigate signaling pathways and target gene expression, we developed different TLR4 (full-length, ΔECD) constructs as an optogenetic tool that allowed precise temporal and reversible stimulation of the TLR4 signaling pathway activation and target gene expression. We first fused the light-oxygen-voltage domain (LOV), a blue light-sensing protein domain isolated from the yellow-green algae *Vaucheria frigida aureochrome 1* (VfAU1-LOV), to the C-terminus of TLR4 (TLR4-LOV). Next, we engineered TLR4-LOV constructs with different truncation variants of the extracellular domain. TLR4 ∆ECD2-LOV contains a deleted LPS/MD2 interaction domain and TLR4 ∆ECD15/21/36-LOV are constructs with different LPS/MD2 interaction domain and dimerization domain deletions (Figure 1a). Previously, the blue-light-sensitive TLR4-LOV chimeric gene stably integrated into a PANC-1 reporter cell line has been shown to be activated by blue light and turned off in the dark in a time- and dose-dependent manner [13] Another article demonstrated that the ectodomain of TLR4 served as an inhibitor to prevent spontaneous, ligand-independent dimerization [25]. To test blue-light-inducible dimerization and downstream signaling of these chimeras, 293Ta cells were transiently co-transfected with NF-κB-Gluc (gaussia luciferase) or AP-1 cis reporter and TLR4, TLR4-LOV, or TLR4 ∆ECD2/15/21/36-LOV constructs using the calcium phosphate precipitation technique (Figure S1a,b) and stimulated with 100 ng/mL LPS, blue light (470 nm), or left untreated for 6 h. Robust NF-κB and AP-1 activation upon blue light exposure could be seen in cells transfected with TLR4-LOV, but not in TLR4 transfected cells. In contrast, LPS stimulation provoked an upregulation of NF-κB and AP-1 in both TLR4-LOV and TLR4 transfected cell lines (Figure 1b,c). Importantly, cells co-transfected with different truncation variants of the TLR4 extracellular domain (TLR4 ∆ECD2/15/21/36-LOV) and reporter plasmids showed high basal NF-κB and AP-1 activation even without stimulation (Figure 1b,c). Additionally, LPS and blue light stimulation triggered an increase in p65 and ERK1/2 phosphorylation in cells transfected with TLR4-LOV, whereas constitutive NF-κB and ERK1/2 signaling could be observed in cells transfected with TLR4 ∆ECD2/15/21/36-LOV, reflected in their high basal activity of unstimulated cells (Figure 1d). No significant difference in NF-κB and ERK1/2 signaling activity could be detected between the different TLR4 ΔECD2/15/21/36-LOV constructs transfected in 293Ta cells (Figure 1b–d).

### 3.2. NF-κB Oscillates in Response to LPS and Blue Light in Engineered Endothelial Cells

**Figure 2:**
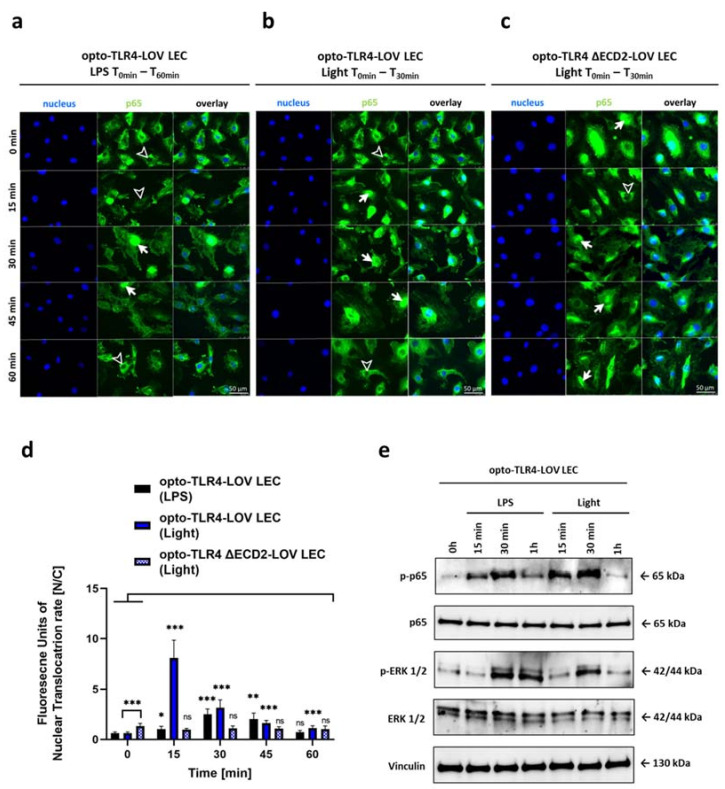
Spatiotemporal kinetics of p65. Intracellular localization of the p65 subunit of NF-κB was detected by immunofluorescence staining in opto-TLR4-LOV LECs treated with (**a**) LPS (100 ng/mL) or (**b**) blue light (470 nm) for 30 min or in (**c**) blue-light-treated opto-TLR4 ΔECD2-LOV. Then, p65 was stained using rabbit anti-p65 as the primary antibody and AF488-conjugated goat anti-rabbit IgG as the secondary antibody (green). Cell nuclei were stained using Hoechst 33342 (blue). Immunofluorescence images were acquired every 15 min for one hour. Arrows indicate prominent p65-NF-κB immunostaining in cell nuclei, while arrowheads display nuclei with deficient p65 labelling. Scale bar = 50 μm. (**d**) Quantitative evaluation of p65-NF-κB nuclear translocation. The mean ratio of relative fluorescence units of stained p65 in the nucleus to the cytoplasm ± standard deviation was graphically displayed in a bar chart (*n =* 6). Post-ANOVA, multiple comparisons relative to the control were performed using Šidák’s test (ns = not significant, * *p* < 0.05, ** *p* < 0.01, *** *p* < 0.001). (**e**) opto-TLR4-LOV LECs were continuously stimulated with LPS (100 ng/mL) or exposed to blue light (470 nm) for 1h. Western blots of whole-cell extracts were probed with antibodies against phospho-p65 (p-p65), p65, phospho-extracellular-signal-regulated kinase 1/2 (p-ERK1/2), ERK1/2, and vinculin as a loading control.

Since the initial experiments suggested that this system was capable of activating TLR4-dependent signaling pathways (Figure 1), we next tested the spatiotemporal expression and localization of TLR4 and the ability of the TLR4-LOV chimeric constructs to induce NF-κB activity in endothelial cells. Therefore, the engineered full-length TLR4-LOV and TLR4 ΔECD2-LOV constructs were stably integrated into human lymphatic endothelial cells (LECs), immortalized by ectopic expression of telomerase reverse transcriptase [14], and into human umbilical vein endothelial cells (HUVECs), using a lentiviral transfection system. Stable integration of full-length TLR4-LOV and TLR4 ΔECD2-LOV was verified by an upregulation of TLR4 mRNA expression levels in engineered target cell lines compared to the wild type, and the change in molecular weight of the TLR4 due to the fusion of the LOV domain or additional truncation of the extracellular domain was verified via western blot analysis (Figure S1c,d,g,h). Temporal TLR4 mRNA expression level analysis of opto-TLR4-LOV LECs during 0-24 h of LPS and blue light treatment revealed a significant elevation after 6 h compared to unstimulated cells. Interestingly, after 24 h of blue light induction, a significant decline in TLR4 mRNA expression compared to unstimulated cells was found, which was not observed in cells treated with LPS. Here, no difference in TLR4 mRNA expression levels compared with the control was detected (Figure S1f). Figure S1e shows microscopic fluorescent images localizing stable integrated full-length TLR4-LOV in LEC. We next tested whether we could measure signaling activity downstream of the optogenetic TLR4 constructs, and sought to investigate the temporal kinetics of p65 and ERK after blue light or LPS induction. When endothelial cells with a stable integrated TLR4-LOV construct were exposed to 470 nm of blue light, a more than seven-fold p65 nuclear localization could be observed within 15 min, whereas nuclear localization could only be seen after 30 min when cells were stimulated with LPS, illustrating the fast activation of the receptor due to the optogenetic approach. After 15 (light) or 30 (LPS) min, a continuous decrease in the nuclear localization of p65 could be found in LPS and blue-light-induced optogenetic cells. In comparison, cells with the truncated version TLR4 ΔECD2-LOV showed a high nuclear translocation even without stimulation (two-fold compared to TLR4-LOV), which slightly decreased 15 min after stimulation, before returning to the basal level within one hour. (Figure 2a–d). To investigate whether the nuclear translocation of opto-TLR4-LOV LECs coincides with p65 and ERK1/2 phosphorylation, we next performed western blot analysis. After blue light or LPS stimulation, a peak phosphorylation of p65 was observed at 15 to 30 min, which could no longer be detected after 1 h. Interestingly, phosphorylation was stronger when cells were illuminated with blue light than when stimulated with LPS. In contrast, strong phosphorylation of ERK1/2 was detected 30 min after blue light or LPS treatment and attenuated after 1 h when cells were exposed to blue light, but not after LPS stimulation.

**Figure 3:**
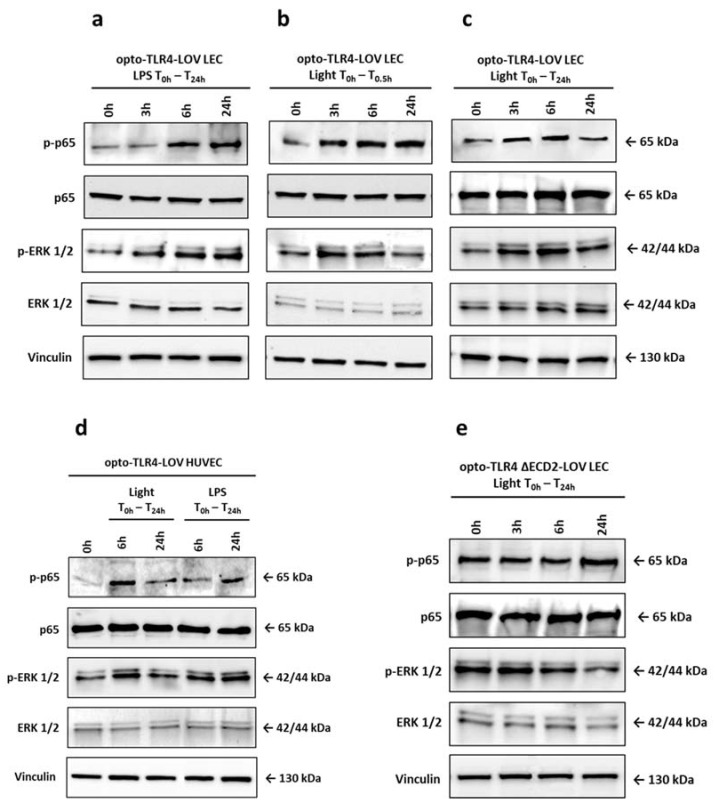
Temporal kinetics of NF-κB and ERK1/2 phosphorylation. (**a**–**e**) Opto-TLR4-LOV LECs were (**a**) continuously stimulated with LPS (100 ng/mL), (**b**) exposed to blue light (470 nm) for 30 min and then put into dark, or (**c**) continuously exposed to blue light (470 nm). (**d**) Opto-TLR4-LOV HUVECs were continuously treated with LPS (100 ng/mL) or illuminated with blue light (470 nm) for 24 h. (**e**) Opto-TLR4 ΔECD-LOV LECs were exposed to blue light (470 nm) for 24 h. Western blots of whole-cell extracts were probed with antibodies against phospho-p65 (p-p65), p65, phospho-extracellular-signal-regulated kinase 1/2 (p-ERK1/2), ERK1/2, and vinculin as a loading control.

Because we detected peak phosphorylation of p65 and ERK1/2 at 15 to 30 min after stimulation with LPS or blue light that leveled off after 1 h, we investigated longer exposure times and if there are differences when cells are illuminated with blue light continuously or only for short time. The opto-TLR4-LOV LECs (Figure 3a,c) and opto-TLR4-LOV HUVECs (Figure 3d) were continuously stimulated with 100 ng/mL LPS or blue light (470 nm), or were exposed to blue light for 30 min and incubated for a further 24 h in the dark (Figure 3b). Protein expression of phospho-p65 and phospho-ERK1/2 was analyzed 3 h, 6 h, and 24 h after the first stimulation with LPS or blue light, respectively. As is shown in Figure 3a–c, strong phosphorylation of p65 could be detected 3 h post-treatment when cells were illuminated with blue light, but not when they were stimulated with LPS. Similarly, ERK1/2 phosphorylation occurred much later when cells were treated with LPS compared to blue light, but they were still strongly activated after 24 h. Upon blue light exposure, peak phosphorylation was seen at 3 to 6 h, which then leveled off by 24 h. Interestingly, continuous blue light exposure caused decreased phosphorylation of p65 after 24 h, which could not be seen in cells that underwent short light or LPS stimulation (Figure 3a–d). In LECs with stable transfected TLR4 ΔECD2-LOV, both p65 and ERK1/2 were already strongly phosphorylated without stimulation. Continuous blue light exposure increased p65 phosphorylation after 24 h, but decreased ERK1/2 phosphorylation over time (Figure 3e).

### 3.3. Blue Light as a Potent Reactivator of NF-κb Signaling

**Figure 4:**
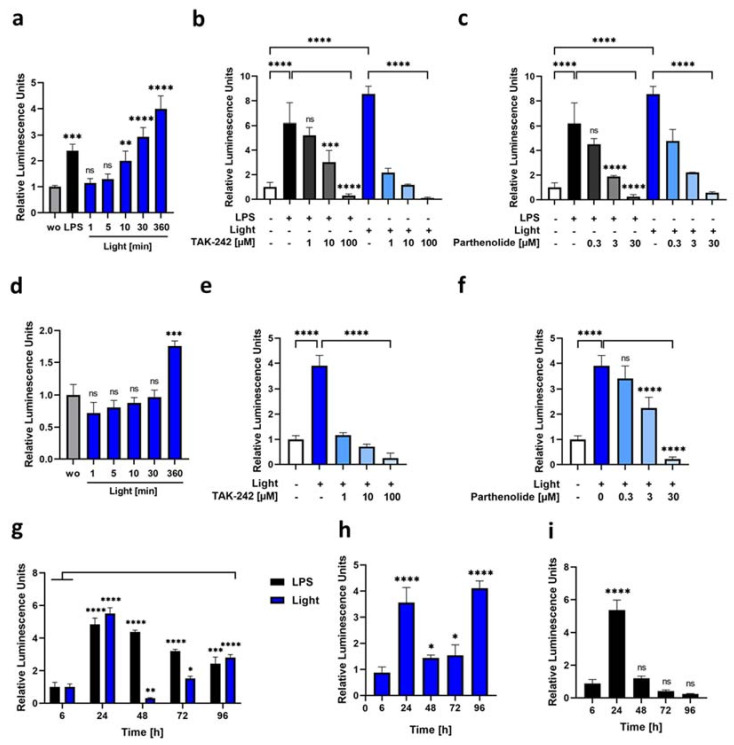
Time- and dose-dependent NF-κB-Gluc reporter activation. (**a**) Opto-TLR4-LOV LECs were continuously stimulated with LPS (100 ng/mL), illuminated with blue light (470 nm) for 6 h, exposed to blue light for 1 min, 5 min, 10 min, 30 min and then put into dark until a total incubation of 6 h was reached, or left untreated (wo). (**b**,**c**) Cells were continuously stimulated with LPS (100 ng/mL) or blue light (470 nm) ± different concentrations of (**b**) TAK-242 (1, 10, 100 µM) or (**c**) parthenolide (0.3, 3, 30 µM), or left untreated for 24 h. (**d**) Opto-TLR4 ΔECD2-LOV LECs were exposed to blue light (470 nm) as in (**a**). (**e**–**f**) Opto-TLR4 ΔECD2-LOV LECs were stimulated with blue light and different concentrations of (**e**) TAK-242 (1, 10, 100 µM) or (**f**) parthenolide (0.3, 3, 30 µM), or left untreated. (**a**–**f**) NF-κB activation was expressed as induction of gaussia luciferase reporter gene and normalized to the cell count. Mean values ± standard deviation were graphically displayed in bar charts (*n* = 6). Post-ANOVA, multiple comparisons relative to the control were performed using Dunnett’s test (ns = not significant, ** *p* < 0.01, *** *p* < 0.001, **** *p* < 0.0001). (**g**) Opto-TLR4-LOV LECs were continuously stimulated with LPS (100 ng/mL) or illuminated with blue light (470 nm), and the medium was changed after every 24, 48, 72 and 96 h post-gaussian-luciferase measurements. (**h**) Cells were exposed to blue light for 30 min at time points 0, 24, 48, 72 and 96 h after Gluc measurement, and then further incubated in the dark up to 24 h. (**i**) Cells were stimulated with LPS (100 ng/mL) for 24 h and medium was changed every 24 h post-luciferase measurements. (**g**–**i**) Mean values ± standard deviation were graphically displayed in bar charts (*n* = 6). Comparative time-curve and reactivation analyses were calculated by subtracting relative luminescence units of the untreated cells at each time point, and normalized to the cell count. Post-ANOVA, multiple comparisons relative to the control were performed using Dunnett’s test (ns = not significant, * *p* < 0.05, ** *p* < 0.01, *** *p* < 0.001, **** *p* < 0.0001).

To investigate the time-dependent activation of the optogenetic receptor constructs, we next stably integrated the NF-κB-TRE-Gluc reporter in both opto-TLR4-LOV LEC full-length and ΔECD constructs using the lentiviral delivery system and selected reporter positive cells using hygromycin B. Next, opto-TLR4-LOV LECs (Figure 4a–c) and opto-TLR4 ΔECD2-LOV LECs (Figure 4d–f) were starved for 24 h with 2% serum overnight and stimulated with 100 ng/mL LPS, illuminated with blue light at 470 nm for 1–360 min or left untreated (wo); NF-κB signaling was quantified by measuring Gluc 6 h after the first stimulation (Figure 4a,d). As depicted in Figure 4a, NF-κB signaling in opto-TLR4-LOV LECs with the integrated full-length construct showed a time-dependent increase peaking (4-fold) at 6 h of continuous blue light induction (Figure 4a). In comparison, the truncated version (ΔECD2) showed no enhanced NF-κB activity compared to the control when cells were illuminated for 1-30 min and only a 1.5-fold increase after 6 h (Figure 4d). To investigate whether this blue-light-induced effect was TLR4-specific, cells were left untreated (dark), treated with LPS, or exposed to blue light for 24 h with 0–100 µM TAK-242 or 0–30 µM parthenolide. As depicted in Figure 4b,c, both the TLR4 inhibitor TAK-242, and the NF-κB inhibitor parthenolide, showed a concentration-dependent downregulation of NF-κB-induced Gluc expression in LPS and blue-light-stimulated opto-TLR4-LOV LECs. Interestingly, although NF-κB-Gluc activity was higher with blue light induction than with LPS, TAK-242 inhibited NF-κB activity more than two-fold when cells were illuminated with blue light, compared to after LPS stimulation, indicating a very specific TLR4 activation by blue light (Figure 4b). Even if the NF-κB reporter activity of the opto-TLR4 ΔECD2-LOV LECs showed only a four-fold elevation after blue light induction, a very specific and dose-dependent inhibition by TAK-242 and parthenolide could be detected (Figure 4e and f). For comparative time-curve and reactivation analyses, we next starved the opto-TLR4-LOV LECs with medium (2% serum) for 16 h, before cells were continuously stimulated (time point 0) with LPS or exposed to blue light for 96 h. The medium was changed every 24 h after the Gluc measurement. As is depicted in Figure 4g, LPS-stimulated cells showed the highest peak after 24 h, which continuously decreased over time, implying that reactivation decreased even after renewed LPS stimulation. Cells illuminated with blue light also peaked after 24 h but could not be reactivated thereafter. After 72 h, there was a slight continuous increase again, but after 96 h, it was half as strong as after 24 h, suggesting that the blue light stimulation exhausted the cell signaling system faster than LPS stimulation. A similar but lower NF-κB-Gluc activity could be observed when cells were only illuminated for 30 min every 24 h (Figure 4h). When cells were stimulated with LPS for 24 h, then incubated for a further 24 in a normal medium, no significant Gluc activity could be detected, even at the time point of 48 h (Figure 4i). In summary, we conclude that the established optogenetic cell lines—opto-TLR4-LOV LECs and opto-TLR4-LOV HUVEC—were very suitable to induce rapid and precise activation of the TLR4. In contrast, due to the truncated extracellular domain, the opto-TLR4 ΔECD2-LOV LECs had a higher basal activity, which also required longer blue light induction to obtain significant additional Gluc activity.

### 3.4. LPS and Blue Light Induce a Temporal Different Pro-Inflammatory Response in Endothelial Cells

**Figure 5:**
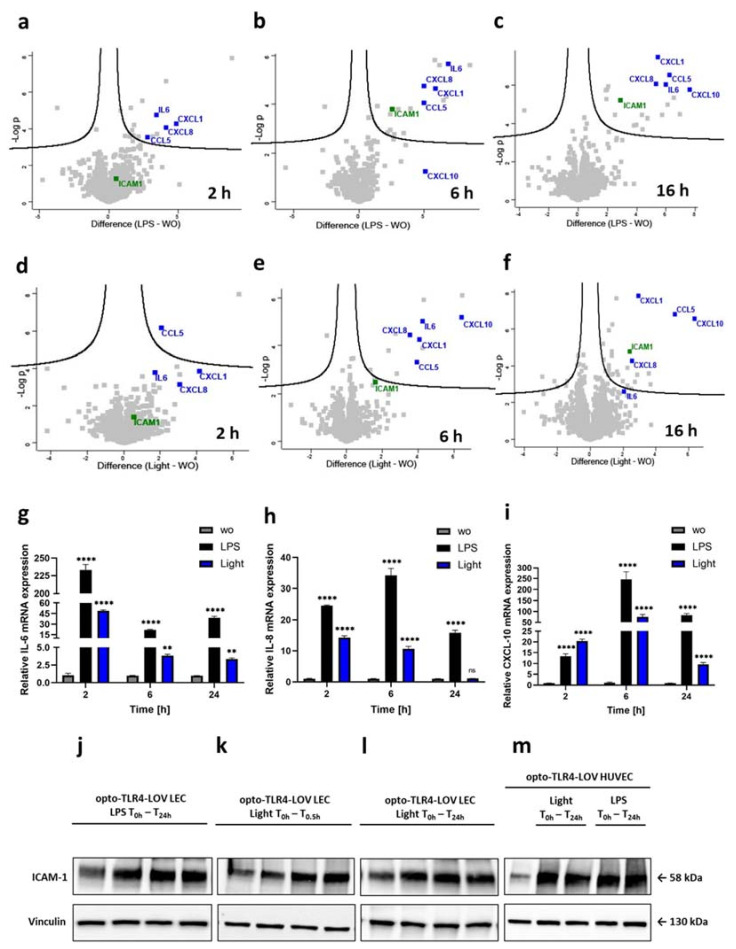
Temporal proteomic and genomic expression analysis of pro-inflammatory mediators. Volcano plots show the regulation of over 1000 proteins in the supernatant of opto-TLR4-LOV LECs after 2 h, 6 h, and 16 h of (**a**–**c**) LPS (100 ng/mL) or (**d**–**f**) blue light (470 nm) stimulation compared to the untreated cells. The difference in LFQ protein abundance between ;reatment and control (wo) (x-axis) was plotted against its significance (-log *p*-value) (y-axis) (*n* = 4). Representative cytokines/chemokines (CXCL-1, CCL-5, IL-6, CXCL-8, and CXCL-10) (blue) and the typical adhesion molecule ICAM-1 (green) were annotated with their corresponding gene name. Relative (**g**) IL-6, (**h**) IL-8, and (**i**) CXCL-10 mRNA expression in opto-TLR4-LOV LECs 2 h, 6 h, and 24 h post-LPS (100 ng/mL) or blue light (470 nm) stimulation. The mRNA target gene expression levels were calculated according to the comparative C_T_ method (2^-ΔΔCT^). Mean values ± standard deviation were graphically displayed in bar charts (*n* = 3). Post-ANOVA, multiple comparisons relative to the control were performed using Dunnett’s test (ns=not significant, ** *p* < 0.01, **** *p* < 0.0001). Opto-TLR4-LOV LECs were (**j**) continuously stimulated with LPS (100 ng/mL), (**k**) exposed to blue light (470 nm) for 30 min and then put into dark, or (**l**) continuously exposed to blue light (470 nm). (**m**) Opto-TLR4-LOV HUVECs were continuously treated with LPS (100 ng/mL) or illuminated with blue light (470 nm) for 24 h. Western blots of whole-cell extracts were probed with antibodies against ICAM-1 and vinculin as a loading control.

Activation of TLR4 and distinct downstream signaling pathways such as NF-κB, AP-1, and IRF3 are known to mediate the transcription of pro-inflammatory cytokine and chemokine genes [26]. Therefore, the supernatant of opto-TLR4-LOV LECs were collected 2 h, 6 h and 16 h after LPS (100 ng/mL) or blue light (470 nm) stimulation, or no treatment (control), and processed for quantitative mass spectrometric analysis. The abundance of over 1000 proteins of four biological replicates at all three time points was determined using a label-free quantification (LFQ) approach (Table S1) and compared to the control (wo) (Figure 5a–f). A significant increase in early pro-inflammatory proteins secreted into the cell supernatant (CXCL-1, CCL-5, IL-6 and CXCL-8) was already found 2 h after LPS stimulation and remained elevated in abundance throughout the 16 h time course. A similar increase in the same pro-inflammatory proteins could be detected after blue light induction. However, protein expression was generally somewhat weaker and therefore not significant for all proteins except CCL-5 after 2 h. In contrast, other proteins such as the chemokine CXCL-10 were found to be highly significant in the supernatant just 6 h after blue light induction, which could not be seen when cells were treated with LPS. The adhesion molecule ICAM-1 was only slightly increased after 2 h of blue light or LPS stimulation, but reached significant elevation after 6 h and 16 h, respectively. Interestingly, after 16 h of continuous blue light illumination, the supernatant protein levels of IL-6 showed a detectable decrease compared to LPS-stimulated cells, whereas the protein levels of other pro-inflammatory mediators such as CXCL-1, CCL-5, CXCL-8, CXCL-10, and the adhesion molecule ICAM-1, remained significantly high after both blue light and LPS stimulation. The temporal accumulation of the pro-inflammatory mediator IL-6, CXCL-8 (IL-8) and CXCL-10 in the supernatant coincided with their augmented mRNA levels measured by RT-qPCR. Here, the target gene expression levels of stimulated opto-TLR4-LOV LECs had already peaked after 2 h (LPS: IL-6; blue light: IL-6, IL-8) or 6 h (LPS: IL-8, CXCL-10; blue light: CXCL-10), and decreased sequentially (Figure 5g–i). Importantly, augmented target gene expression of IL-6, IL-8, and CXCL-10 after 2 h of LPS/blue light stimulation could be confirmed in HUVECs with stable integrated TLR4-LOV (Figure S2a–c). Interestingly, upregulation of these pro-inflammatory genes was also observed in opto-TLR4 ΔECD2-LOV LECs after 2 h of LPS or blue light treatment, which then leveled off after 6 h and increased again after 24 h (Figure S2e–g). The adhesion molecule ICAM-1 is a main driver for leukocyte adhesion and trans-endothelial migration. It is expressed at a low level on the vascular endothelium and is increased by inflammatory stimuli [27]. Therefore, temporal ICAM-1 protein expression was analyzed upon LPS or blue light treatment during 0–24 h in engineered endothelial cell lines. Opto-TLR4-LOV LECs and opto-TLR4-LOV HUVECs were continuously stimulated with LPS (100 ng/mL) or blue light (470 nm), or opto-TLR4-LOV LECs were additionally exposed to blue light for 30 min and incubated for a further 24 h in the dark. Protein expression of ICAM-1 was analyzed 0–24 h after stimulation with LPS or blue light, respectively. Strong ICAM-1 expression could be detected 3–6 h post-LPS or blue light treatment, which then remained elevated throughout the 24 h time course (Figure 5j–m). In LECs with stable integrated TLR4 ΔECD2-LOV, high basal (0 h) ICAM-1 expression was found, which was slightly decreased after 24 h of continuous blue light treatment (Figure S2h).

### 3.5. Blue Light and LPS Stimulation Promote Chemotactic Migration, Endothelial Barrier Disruption and Trans-Endothelial Migration

**Figure 6:**
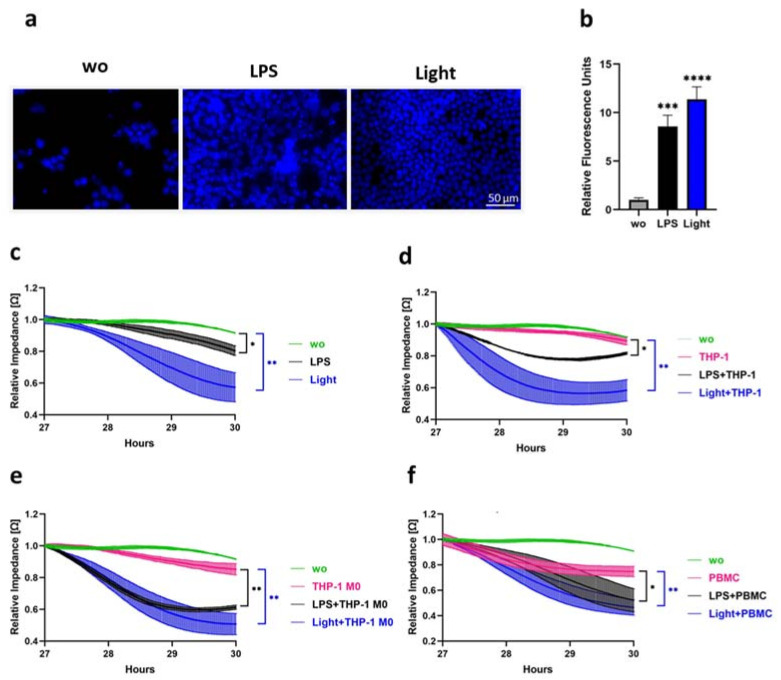
Chemotactic and trans-endothelial migration. (**a**,**b**) THP-1 cells stained with Hoechst 33342 were seeded in the upper chamber of (**a**) Transwell^®^ 24-well permeable supports or (**b**) Transwell^®^ 96-well permeable supports and allowed to migrate through the 5 µm pore-size filters for 2 h towards endothelial medium of opto-TLR4-LOV LECs stimulated with LPS (100 ng/mL), illuminated with blue light (470 nm), or left untreated for 6 h. Migrated THP-1 cells were (**a**) visualized by fluorescence microscopy or (**b**) measured by multiplate reader. (**a**) Scale bar = 50 µm. (**b**) Bar charts representing mean values ± standard deviation of the well scan of relative fluorescence units (*n* = 3). Post-ANOVA, multiple comparisons relative to the control were performed using Dunnett’s test (*** *p* < 0.001, **** *p* < 0.0001). (**c**–**f**) LPS-, blue light- and monocytic-cell-line-induced EC monolayer breakdown in opto-TLR4-LOV LECs. ECs were seeded onto ECIS arrays (96W20idf PET) and allowed to grow to a monolayer before being (**c**) treated with LPS (100 ng/mL), blue light (470 nm), or left untreated. Addition of 50,000 (**d**) THP-1 cells/well, (**e**) THP-1 M0 cells/well, or (**f**) PBMCs/well. (**c**–**f**) Changes in endothelial monolayer resistance, which is proportional to endothelial barrier function, were recorded in real time using the ECIS system (9600Z) and mean values ± standard deviation (n = 8) were plotted in a time-curve diagram. Post-ANOVA, multiple comparisons relative to the control 3 h after treatment (T_30h_) were performed using Dunnett’s test (* *p* < 0.05, ** *p* < 0.01).

To monitor key cellular processes that play an important role during inflammation, different functional assays were performed to measure the chemoattraction of THP-1, barrier function, and the transmigration of leukocytes through the endothelial cell monolayer. To determine whether opto-TLR4-LOV LECs induced the chemoattraction of monocytic cells after blue light or LPS stimulation, THP-1 cells were stained with Hoechst 33342 and seeded into the upper chamber of Transwell^®^ 96-well permeable supports and Transwell^®^ 24-well permeable supports and allowed to migrate through the 5 µM pore-size filters for 2 h towards a medium of opto-TLR4-LOV LECs stimulated with LPS (100 ng/mL), blue light (470 nm), or left untreated for 6 h, in the lower chambers of the transwells. THP-1 cells that migrated through the filter of the 96-well permeable supports were quantified by measuring relative fluorescence units with a plate reader in a well scan manner, whereas THP-1 cells that migrated through the filter of 24-well permeable supports were visualized by fluorescence microscopy. As such, we found that the media of LPS- and blue-light-treated opto-TLR4-LOV LECs triggered a chemotactic response on THP-1 cells, with the highest migration detected with blue light (Figure 6a,b). To study whether LPS and blue light induce the breakdown of the EC monolayer and infiltration of monocytes in opto-TLR4-LOV LECs and opto-TLR4-LOV HUVEC, we applied the ECIS technology. Consequently, endothelial monolayer resistance, which is proportional to endothelial barrier function, can be monitored in real time by means of measuring the impedance over time. First, opto-TLR4-LOV LECs and opto-TLR4-LOV HUVECs were cultured onto ECIS arrays and allowed to grow to a monolayer before they were challenged with LPS (100 ng/mL), blue light (470 nm), and/or with different differentiated monocytic cell lines (THP-1 cells, THP-1 M0 cells) and PBMCs (50,000 cells/well). Endothelial barrier function was subsequently assessed by continuous resistance measurement. LPS and blue light treatment significantly enhanced the disruption of the opto-TLR4-LOV LEC and opto-TLR4-LOV HUVEC monolayers (Figure 6c and Figure S3a). Trans-endothelial migration of the monocytic cell lines or PBMCs through the opto-TLR4-LOV LEC and opto-TLR4-LOV HUVEC monolayers could be increased with additional LPS or blue light treatment. (Figure 6d–f and Figure S3b). Similar effects upon illumination were observed using opto-TLR4 ∆ECD2-LOV LECs (Figure S3c–f). These results clearly show that blue light, like LPS, promotes not only the chemotactic but also trans-endothelial migration of monocytic cell lines and PBMCs.

## 4. Discussion

This study aimed to examine how TLR4 activation is processed downstream in endothelial cells, focusing mainly on the temporal organization of signaling pathways and target gene expression.

Carrying out these measurements requires fast and precise activation of the receptor, which is difficult to achieve with ligands such as LPS due to the often slow and poorly controllable specific and non-specific binding to receptors and other surface molecules [28,29]. Furthermore, there are hardly any processes in which LPS can be isolated with high purity, triggering various cell activations [30]. Therefore, we engineered new light-oxygen-voltage (LOV)-sensing domain-based optogenetic endothelial cell lines with full-length TLR4 (opto-TLR4-LOV LEC and HUVEC) to allow precise temporal and reversible activation of TLR4 signaling pathways and target gene expression. TLR4 homodimerization in the engineered ECs relies on the LOV domain isolated from the yellow-green algae *Vaucheria frigida aureochrome 1* (VfAU1-LOV) fused C-terminally to TLR4. VfAU1-LOV noncovalently binds a flavin chromophore, which upon blue light (470 nm) absorption, initiates a photochemical reaction leading to the formation of a covalent adduct between the conserved cysteine and the flavin ring. The result is a conformational change that allows the dimerization of the LOV domains [31]. Since flavin nucleotides (FMN) are readily available in mammalian cells, no addition of exogenous molecules is required. Previously, we have described a technically similar engineered pancreatic adenocarcinoma cell line (opto-TLR4 PANC-1) that allows time- and voltage-dependent TLR4 activation by blue light, which can be switched off again in the dark. Low intensity blue light (8 V) was shown to be sufficient for the activation of the engineered TLR4-LOV construct and does not produce phototoxicity [13].

In this study, we demonstrated that 293Ta transiently transfected with TLR4-LOV but not with TLR4 lacking the LOV domain was able to activate NF-κB and AP-1 reporter activities upon blue light exposure. In contrast, NF-κB and AP-1 expression was significantly elevated after LPS stimulation in both TLR4 and TLR4-LOV transfected cells. Additionally, the newly engineered optogenetic endothelial cell lines were found to initiate TLR4-specific signaling events much faster and stronger upon illumination with blue light compared to the stimulation with LPS. It is well known that TLR4 signaling triggers the translocation of the pro-inflammatory transcription factor NF-κB from the cytoplasm into the nucleus to initiate gene transcription. In the nucleus, the newly synthesized protein IκBα (inhibitor of nuclear factor kappa B) deactivates NF-κB and facilitates its export back to the cytoplasm [32,33]. Oscillation of NF-κB in and out of the nucleus upon stimulation is reported to contribute considerably to the pattern of inflammatory gene expression [34]. Consistent with other studies [35,36], we found that LPS stimulation causes rapid (30 min) and transient translocation of p65 into the nucleus in opto-TLR4-LOV LECs. However, blue light illumination provoked an even faster and stronger p65 nuclear translocation (seven-fold and within 15 min) compared to LPS treatment, which also declined within an hour. Moreover, peak phosphorylation of p65 and ERK1/2 in opto-TLR4-LOV endothelial cells was measured 15 to 30 min after LPS or blue light stimulation, and again after 3 h and 6 h when cells were stimulated with light or LPS, respectively. On the other hand, we also found that the light-induced TLR4 signaling system was exhausted much quicker compared to LPS treatment. Studies have highlighted LPS as an agonist for receptors other than TLR4, including Toll-like receptor 2 (TLR2) [37], nucleotide-binding oligomerization domain (NOD)-like receptor 1 [38], and retinoic acid-inducible gene 1 (RIG-1)-like receptor [39], all of which are reported to induce NF-κB activity. TLR4–TLR2 cross talk was reported to be mediated by MyD88, resulting in the amplification and stable expression of NF-κB and ICAM-1 [40]. In addition, the cytosolic receptors NOD1 and RIG-1 are described to be activated upon internalization of LPS [41,42,43]. The resulting downstream signal transduction of the NF-κB and MAPK pathways amplifies the transcription of inflammatory cytokines and chemokines, which are also induced by TLR4 signaling [44,45]. Notably, RIG-1 was found to be a key factor in the autoloop cascade for late MyD88-independent activation of NF-κB, thereby maintaining sustained secretion of pro-inflammatory mediators [43,46]. Here, we show that continuous light exposure decreased phosphorylation of p65 after 24 h, which could not be seen when cells were permanently LPS stimulated, indicating a specific activation of TLR4 by light. Furthermore, by performing reporter analysis, we showed that NF-κB activation was much less repressed after TAK-242 (TLR4 inhibitor) treatment than after light induction. This is due to the binding of LPS to additional receptors other than TLR4 that amplify the NF-κB signal. In addition to the fast light-induced depletion, we were also able to demonstrate that the signaling pathways can be reactivated after a certain period, as demonstrated with the NF-κB-TRE-Gluc reporter assay. Here, exhaustion of the NF-κB signaling was observed between 48 and 72 h, with both continuous and recurrent illumination for 30 min every 24 h. Blue-light-induced TLR4 and NF-κB reactivation could be detected again after 72 h (continuous illumination) and 96 h (30 min every 24 h), respectively. In contrast, with continuous LPS treatment, NF-κB decreased slightly over the time course (4.5–2.5-fold), but remained relatively high even after 96 h. This phenomena has been studied extensively in monocytes and macrophages [44,47], but has also previously been reported in endothelial cells [48].

Panter and Jerala (2011) have clearly demonstrated that the ectodomain of TLR4 prevents constitutive receptor activity, since truncation of the TLR4 ectodomain from its N-terminus ultimately resulted in persistent active receptor variants [25]. They further showed that the ectodomain exhibits strong regulatory properties enabling a controlled ligand receptor activation by providing proper localization and inhibition of spontaneous, ligand-independent receptor dimerization. Consistent with this study, we found that transient transfection of different TLR4 ΔECD-LOV variants compared with the TLR4-full length-LOV into 293Ta were already relatively strongly activated prior to light stimulation. Here, no difference in NF-κB activity and p65 or ERK1/2 phosphorylation could be detected between TLR4 ΔECD2-LOV (with a deleted LPS/MD2 domain) and ΔECD15/21/36-LOV (with a deleted LPS/MD2 and dimerization domain). Stable integration of TLR4 ΔECD2-LOV into LECs revealed a high basal activity with fast depletion of the cell signaling system upon additional blue light stimuli, as evidenced by p65 and ERK1/2 phosphorylation, and nuclear translocation of NF-κB. NF-κB reporter activity and mRNA gene expression of pro-inflammatory genes were found to exert a slight activation potential with fast depletion of the TLR4 signaling system. Of note, it was also observed in TLR4 ΔECD2-LOV that the mRNA gene expression of pro-inflammatory genes (IL-6, IL-8, and CXCL-10) was elevated after 2 h of light induction, but it was depleted 6 h later and could be reactivated after 24 h.

Because the endothelium forms a strong barrier that separates circulating blood from tissue, access to potential sites of infection requires the expression of different genes [49]. By performing functional assays using opto-TLR4-LOV LECs and HUVEC, we were able to show that TLR4 stimulation with blue light promoted chemotaxis of THP-1 cells, disruption of the EC monolayer, and transmigration that was faster and stronger compared to treatment with LPS. A higher EC-monolayer break-up and transmigration could also be seen in the TLR4 ΔECD2-LOV after light induction. Of note, proteomic analysis of opto-TLR4-LOV LECs revealed a temporal elevation of secreted disintegrin and metalloproteinase with thrombospondin motifs 4 (ADAMTS4) and the matrix metalloproteinase 10 (MMP10), both contributing to the degradation of the extracellular matrix and thus facilitating trans-endothelial migration [50,51,52,53].

Since LPS activates receptors other than TLR4, as already described, we assumed higher transcription rates of pro-inflammatory genes. As such, CXCL-1, CXCL-8, and IL-6 were found in higher concentration in the supernatant after 2 h of LPS stimulation compared to light induction. We further found that the protein level of IL-6 in the supernatant showed a detectable decrease after 16 h of continuous blue light illumination, which was not seen with LPS stimulation. However, a significant increase in known secreted pro-inflammatory mediators (CXCL-1, CCL5, IL-6, and CXCL-8) was observed 6 h after LPS or blue light stimulation, consistent with published studies on LPS-activated endothelial cells [54,55,56]. In addition, a strong and persistent expression of ICAM-1 as a transmembrane protein and secretion has been found in activated endothelial cells [48,57,58]. Time-course analysis of target mRNA expression levels of IL-6, IL-8 and CXCL-10 confirmed a generally stronger and more persistent expression of pro-inflammatory mediators when optogenetic cells were treated with LPS compared to light exposure. Interestingly, the chemokine CXCL-10 was found to be highly significant in the supernatant as early as 6 h after light induction, which was not seen when cells were treated with LPS. In addition, CXCL-10 mRNA expression in optogenetic endothelial cell lines was already higher when exposed to blue light for 2 h compared to LPS treatment. A recent temporal proteomic analysis of pro-inflammatory mediators in LPS-induced THP-1 cells revealed a significant upregulation of CXCL-10 6 h post-stimulation [59].

## 5. Conclusions

In summary, we conclude that the newly-engineered optogenetic cell lines elicit faster and more precise TLR4 activation under blue light illumination compared to LPS stimulation. Gene expression of pro-inflammatory target genes is, however, reduced and less persistent. In contrast, due to the truncated extracellular domain, opto-TLR4 ΔECD2-LOV LECs show a high basal activity with fast depletion of the cell signaling system upon additional blue light stimuli.

Attaining deeper insights into the molecular and regulatory mechanisms of pro-inflammatory TLR4 signaling events in endothelial cells with spatiotemporal precision will expedite the establishment of novel therapeutic strategies beneficial for the treatment of sepsis and chronic inflammatory diseases.

## Figures and Tables

**Table 1:** List of antibodies for Western blotting. Cell Signaling (Danvers, MA, USA), Santa Cruz (Dallas, TX, USA), US Biological (Salem, MA, USA), Abcam (Cambridge, UK). ICAM-1, intracellular adhesion molecule 1; ERK1/2, extracellular-signal regulated kinase; HRP, horseradish peroxidase; p, phosphorylated.

Target	Cat#	Dilution	Diluent	Host	Company
Vinculin	sc-73614 HRP	1:200	PBS-T (0.1%)	Mouse	Santa Cruz Biotechnology
TLR4	042879	1:1000	PBS-T (0.1%)	Rabbit	US Biological
p-p65	sc-136548 HRP	1:200	PBS-T (0.1%)	Mouse	Santa Cruz Biotechnology
p65	ab16502	1:1000	PBS-T (0.1%)	Rabbit	Abcam
ICAM-1	ab109361	1:1000	PBS-T (0.1%)	Rabbit	Abcam
p-ERK1/2	9101	1:1000	PBS-T (0.1%)	Rabbit	Cell Signaling
ERK1/2	9102	1:1000	PBS-T (0.1%)	Rabbit	Cell Signaling
HRP anti-rabbit	7074	1:1000	PBS-T (0.1%)	Goat	Cell Signaling

## Data Availability

Data presented in this study are available on request from the corresponding authors.

## Supplementary Materials

The following supporting information can be downloaded at: https://www.mdpi.com/article/10.3390/cells12050697/s1, Figure S1: TLR4-LOV construct expression analysis. Figure S2: Pro‐inflammatory gene expression analysis. Figure S3: Pro-inflammatory gene expression analysis. Figure S4: Trans-endothelial migration analysis. Table S1: Temporal proteomics data.
