# Supplementary material for: Comparative and Temporal Characterization of LPS and Blue-Light-Induced TLR4 Signal Transduction and Gene Expression in Optogenetically Manipulated Endothelial Cells
Source: Cells. 2023 Feb 22;12(5):697. doi: 10.3390/cells12050697 (PMC10000987; doi:10.3390/cells12050697)
Supplement: Supplementary file 1 [file cells-12-00697-s001.zip › cells-2127609-supplementary.pdf]

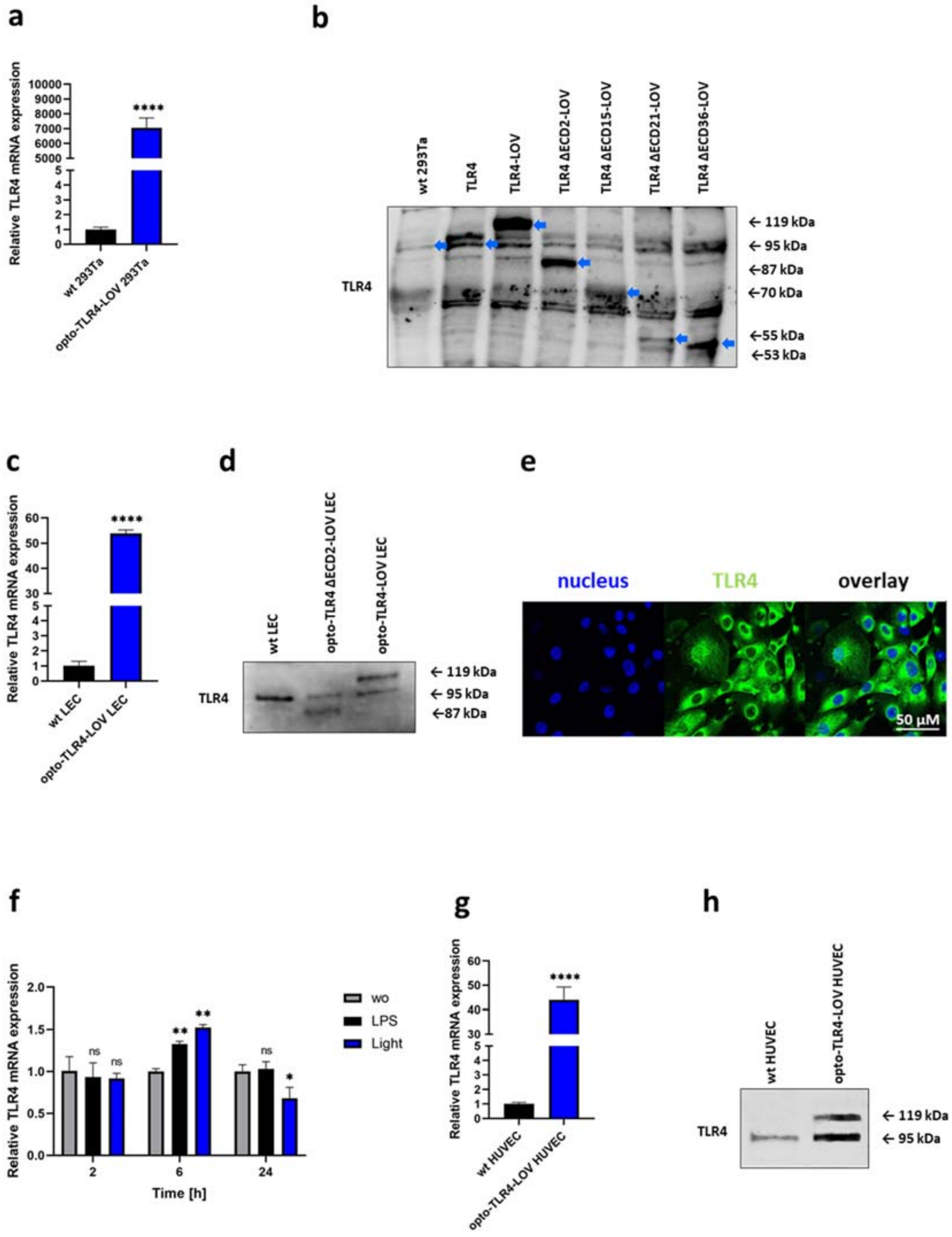

Figure S1. TLR4-LOV construct expression analysis. Relative TLR4 mRNA expression of (**a**) wild-type 293Ta cells or 293Ta cells transiently transfected with the TLR4-LOV construct, (**c**) wild-type LEC or LEC with stable incorporated TLR4-LOV construct, (**f**) opto-TLR4-LOV LEC 2 h, 6 h and 24 h post LPS (100 ng/mL) or blue light (470 nm) stimulation, or no treatment, and (**g**) wild-type HU-VEC or HUVEC with stable incorporated TLR4-LOV construct was calculated according to the com-parative CT method (2-ΔΔCT). **(a, c, g**) Mean values ± standard deviation were graphically dis-played in bar charts (n=3) and relatively compared to the control using Student's t-test (ns=not significant, *p<0.05, **p<0.01, ***p<0.001, ****p<0.0001). (**b, d, h**) Western Blot analysis of TLR4 protein expres-sion of (**b**) wild-type 293Ta cells, 293Ta cells transiently transfected with the TLR4, TLR4-LOV or TLR4 ΔECD2/15/21/36-LOV construct using the calcium phosphate precipitation technique, (**d**) wild-type LEC or LEC with stable incorporated TLR4-LOV or TLR4 ΔECD2-LOV construct using a lentiviral transfection system, and (**h**) wild-type HUVEC or HUVEC with stable incor-porated TLR4-LOV construct using a lentiviral transfection system. (**e**) Representative confocal laser scanning mi-cros-copy showing TLR4 localization of opto-TLR4-LOV LEC. TLR4 was stained using rabbit anti-TLR4 as the primary antibody and AF488 conjugated goat anti-rabbit IgG as the secondary antibody (green). Cell nuclei were stained using Hoechst 33342 (blue). Scale bar=50 μm.

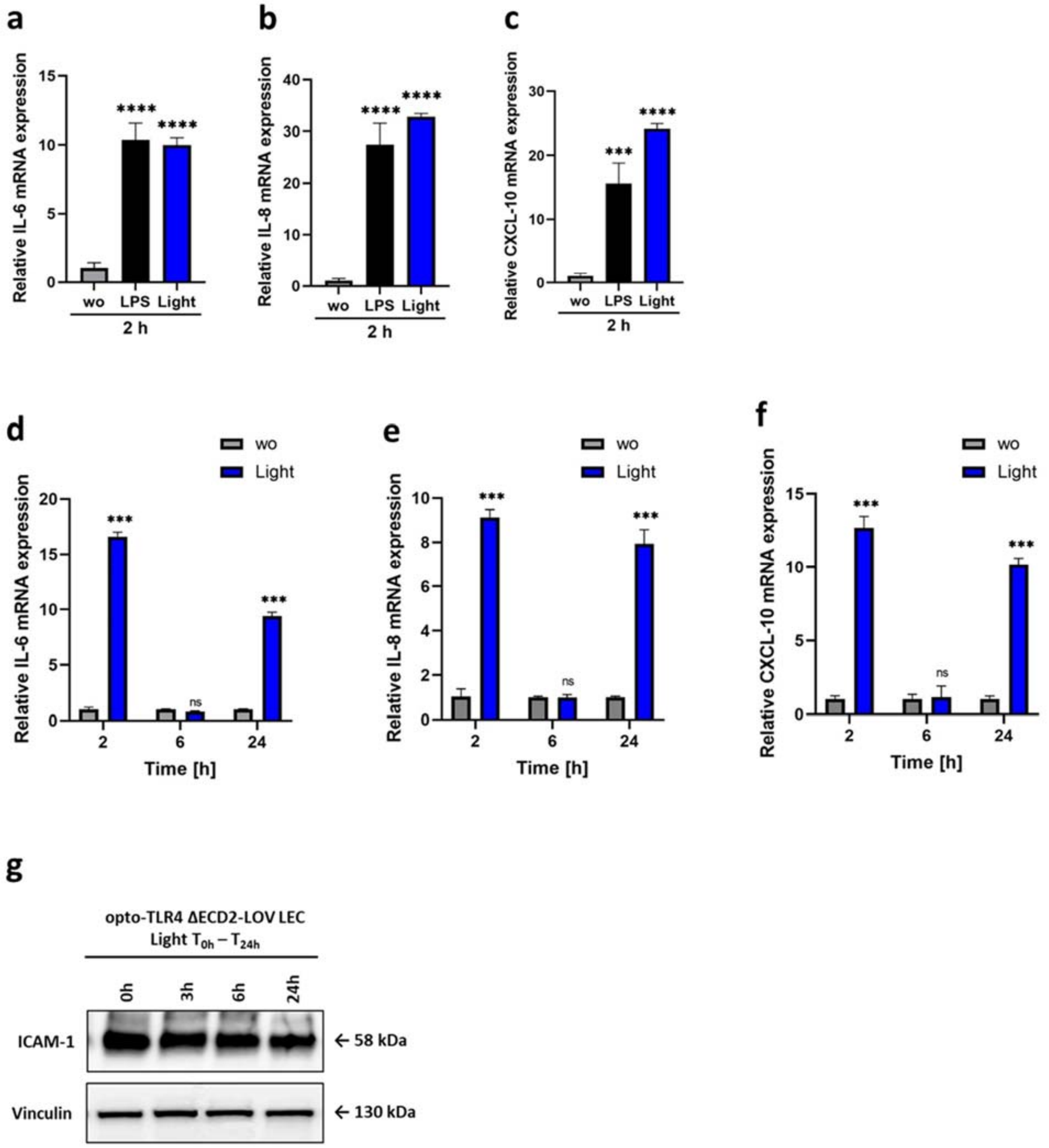

Figure S2. Pro-inflammatory gene expression analysis. Relative IL-6, IL-8 and CXCL-10 mRNA expression in (**a-c**) opto-TLR4-LOV HUVEC 2 h post and (**d-f**) opto-TLR4 ΔECD2-LOV LEC 2 h, 6 h post blue light (470 nm) stimulation. The mRNA target gene expression levels were calculated according to the comparative CT method ($2^{-\Delta\Delta CT}$). Mean values ± standard deviation were graphically displayed in bar charts (n=3). Post-ANOVA multiple comparisons relative to the control were per-formed using Dunnett's test (ns=not significant, **p<0.01, ***p<0.001, ****p<0.0001). (**g**) Western Blots of opto-TLR4 ΔECD2-LOV LEC illuminated with blue light (470 nm) for 0-24 h. Whole-cell extracts were probed with antibodies against ICAM-1 and vinculin as a loading control.

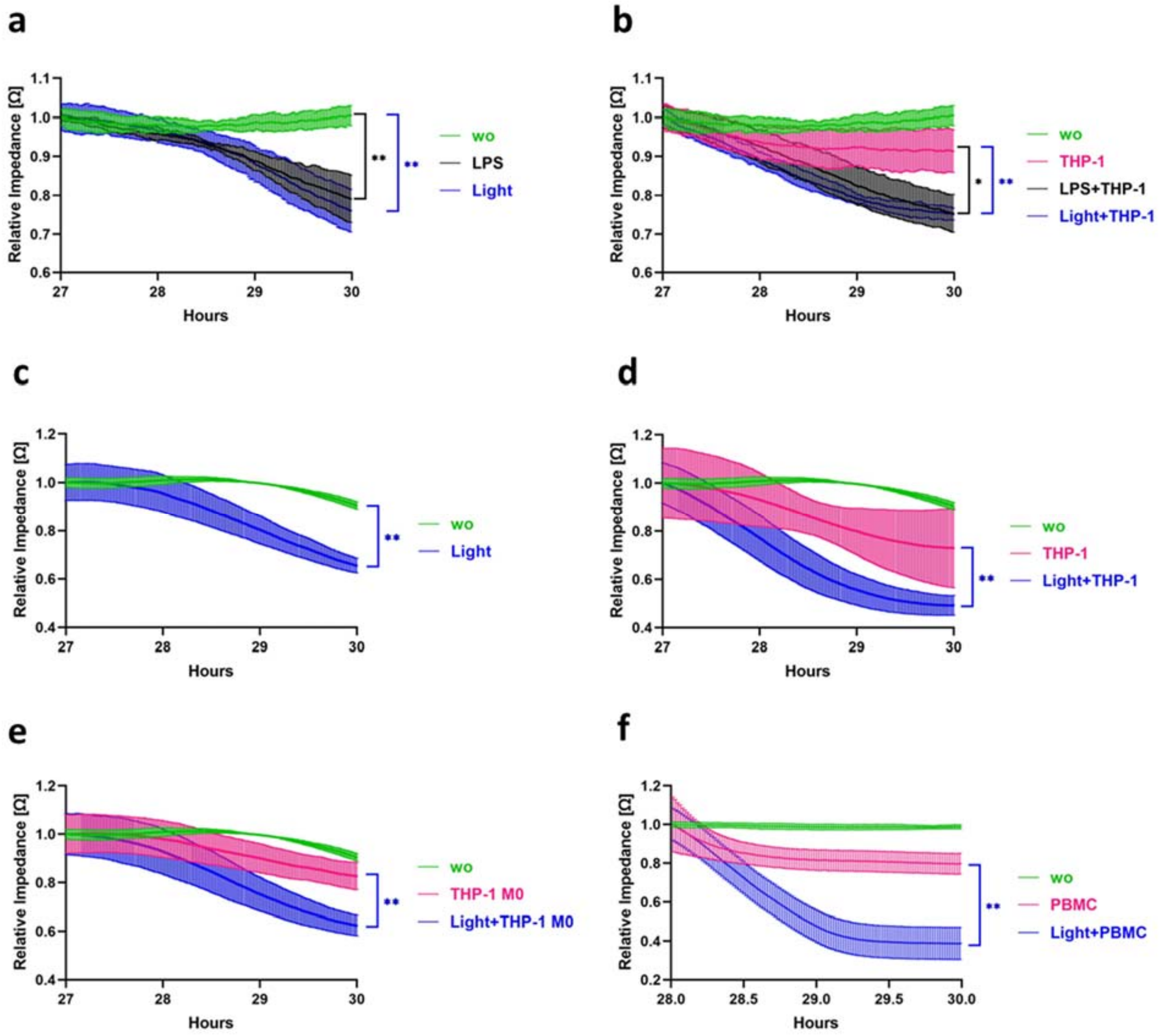

Figure S3. Trans-endothelial migration analysis. LPS-, blue-light- and monocytic-cell-line-induced EC monolayer breakdown in (a, b) opto-TLR4-LOV HUVEC and (c-f) opto-TLR4 ΔECD2-LOV LEC. ECs were seeded onto ECIS arrays (96W20idf PET) and allowed to grow to a monolayer before being treated with (a) LPS (100 ng/mL), blue light (470 nm) or left untreated or (c) blue light (470 nm) or left untreated. Addition of 50,000 (**b, d**) THP-1 cells/well, (**e**) THP-1 M0 cells/well, or (**f**) PBMCs/well. (**a-f**) Changes in endothelial monolayer resistance, which is proportional to endothelial barrier function, were recorded in real time using the ECIS system (9600Z) and mean values ± standard deviation (n=8) were plotted in a time-curve diagram. Post-ANOVA multiple comparisons relative to the control three hours after treatment (T_{30h}) were performed using Dunnett's test (*p<0.05, **p<0.01).

Table S1. Temporal proteomics data. Protein level quantification of supernatant of opto-TLR4-LOV LEC 2 h, 6 h and 16 h post LPS (100 ng/mL) or blue light (470 nm) stimulation. List of LFQ intensities and significant regulations are extracted from Perseus. Missing values have been re-placed by imputation using a normal distribution with a width of 0.3 and a down shift of 1.8 to enable statistical testing. Volcano plots and the respective significant proteins were determined using a two-sided T-test with an FDR=0.05 and an S0=0.1. Significantly regulated proteins are marked in the corresponding column and the -log(p-value) and the difference are stated for each pairwise comparison of WO vs. LPS. WO vs. light and LPS vs. light for 2, 6 and 16 hours. WO=without treatment.
